# AMPKα2 promotes tumor immune escape by inducing CD8+ T-cell exhaustion and CD4+ Treg cell formation in liver hepatocellular carcinoma

**DOI:** 10.1186/s12885-024-12025-y

**Published:** 2024-03-01

**Authors:** Yan Ouyang, Yan Gu, Xinhai Zhang, Ya Huang, Xianpeng Wei, Fuzhou Tang, Shichao Zhang

**Affiliations:** 1 Key Laboratory of Infectious Immune and Antibody Engineering of Guizhou Province, Engineering Research Center of Cellular Immunotherapy of Guizhou Province, Guizhou Medical University, Guiyang, China; 2 Immune Cells and Antibody Engineering Research Center of Guizhou Province, Key Laboratory of Biology and Medical Engineering, Guizhou Medical University, Guiyang, China

**Keywords:** AMPKα2, Cell communication, CD8+ T-cell exhaustion, Tumor immune escape, Immunotherapy, Liver hepatocellular carcinoma

## Abstract

**Background:**

Adenosine monophosphate-activated protein kinase (AMPK) is associated with the development of liver hepatocellular carcinoma (LIHC). AMPKα2, an α2 subunit of AMPK, is encoded by *PRKAA2*, and functions as the catalytic core of AMPK. However, the role of AMPKα2 in the LIHC tumor immune environment is unclear.

**Methods:**

RNA-seq data were obtained from the Cancer Genome Atlas and Genotype-Tissue Expression databases. Using the single-cell RNA-sequencing dataset for LIHC obtained from the China National Genebank Database, the communication between malignant cells and T cells in response to different *PRKAA2* expression patterns was evaluated. In addition, the association between *PRKAA2* expression and T-cell evolution during tumor progression was explored using Pseudotime analysis, and the role of *PRKAA2* in metabolic reprogramming was explored using the R “*scMetabolis*” package. Functional experiments were performed in LIHC HepG2 cells.

**Results:**

AMPK subunits were expressed in tissue-specific and substrate-specific patterns. *PRKAA2* was highly expressed in LIHC tissues and was associated with poor patient prognosis. Tumors with high *PRKAA2* expression displayed an immune cold phenotype. High *PRKAA2* expression significantly promoted LIHC immune escape. This result is supported by the following evidence: 1) the inhibition of major histocompatibility complex class I (MHC-I) expression through the regulation of interferon-gamma activity in malignant cells; 2) the promotion of CD8+ T-cell exhaustion and the formation of CD4+ Treg cells in T cells; 3) altered interactions between malignant cells and T cells in the tumor immune environment; and 4) induction of metabolic reprogramming in malignant cells.

**Conclusions:**

Our study indicate that *PRKAA2* may contribute to LIHC progression by promoting metabolic reprogramming and tumor immune escape through theoretical analysis, which offers a theoretical foundation for developing *PRKAA2*-based strategies for personalized LIHC treatment.

**Supplementary Information:**

The online version contains supplementary material available at 10.1186/s12885-024-12025-y.

## Introduction

Liver hepatocellular carcinoma (LIHC) is the main histological subtype of primary liver cancer. LIHC is highly aggressive and therapeutic options are limited. Thus, the prognosis for patients with LIHC is very poor [1, 2]. The current drugs for treating LIHC include sorafenib, lenvatinib, and regorafenib [3], which are multitarget tyrosine kinase inhibitors, and atezolizumab, pembrolizumab, nivolumab, and ipilimumab [4], which are immunotherapeutic agents. Although these drugs have achieved significant success in the treatment of LIHC, treatment benefits are limited to a small subset of patients [5, 6]. LIHC is an extremely heterogeneous tumor, which limits the efficacy of cancer therapies [7]. Therefore, new effective diagnostic, prognostic, and therapeutic biomarkers based on single-cell analyses are urgently needed to develop personalized therapeutic strategies against LIHC.

Adenosine monophosphate-activated kinase (AMPK), a serine/threonine protein kinase, consists of AMPKα (catalytic core; α1 or α2), AMPKβ and AMPKγ (regulatory units; β1 or β2, and γ1, γ2, or γ3) [8]. AMPK activates or inhibits metabolic-related pathways in response to changes in intracellular AMP/ATP ratios [9]. Theoretically, human AMPK can form 12 different isoforms, depending on the combination of subunit subtypes. The expression of heterotrimeric complexes of AMPK varies widely in mammalian eukaryotic cells [10]. AMPK subunits are distributed differently in tissues and organs, and the distribution may be related to the regulation of tissue-specific target molecules. In addition, many AMPK substrates are distributed in cells and tissues [11]. However, the tissue and substrate specificity of AMPK complexes are unclear.

AMPKα2 is encoded by the *PRKAA2* gene. *PRKAA2* plays important roles in both tumor initiation and progression, including the regulation of mTOR kinase activity and the maintenance of NADPH levels [12, 13]. Changes in *PRKAA2* expression have been linked to the occurrence, development, and prognosis of multiple tumor types, including breast cancer, ovarian cancer, gastric cancer, kidney cancer, and liver hepatocellular carcinoma [14–16]. Thus, *PRKAA2* is a potential target for new therapeutic strategies. *PRKAA2* may influence tumor immunity in some cancer types. For instance, Zhang et al. constructed a risk model using *PRKAA2* and eight other genes. The resulting risk scores were closely linked to immunotherapy responses in patients with head and neck squamous cell carcinoma [17]. Stromal cells, which are the most active cell type in the tumor microenvironment (TME), predominantly consist of endothelial cells, epithelial cells, fibroblasts, and immune cells, including T cells, B cells, neutrophils, and macrophages [18]. Dynamic interactions between these cells are a major determinant of tumor pathophysiology [19]. Therefore, single-cell analysis is needed to explore the functional roles of *PRKAA2* in the TME.

We demonstrated that AMPK subunits exhibit tissue-specific expression patterns and determine substrate specificity and physiological function. *PRKAA2*, which codes for the catalytic core of AMPK, was expressed at significantly high levels in LIHC, and expression of *PRKAA2* was associated with poor prognosis. Thus, our studies focused on *PRKAA2*. The roles of *PRKAA2* in LIHC and the relationship between *PRKAA2* and the LIHC tumor immune microenvironment and malignant cell metabolism were explored. *PRKAA2* may contribute to LIHC progression by inducing metabolic reprogramming of malignant cells and promoting immune escape of tumor cells. Moreover, patients with high *PRKAA2* expression displayed an immune cold phenotype, while tumors with low *PRKAA2* expression exhibited the opposite immune characteristics. Our results provide a theoretical foundation for developing *PRKAA2*-based strategies for individualized treatment of patients with LIHC.

## Methods

### Data acquisition

RNA-seq data for 31 normal tissues and LIHC samples were obtained from the Genotype-Tissue Expression (GTEx, https://gtexportal.org/home/) and the Cancer Genome Atlas (TCGA, https://www.cancer. gov/tcga) databases, respectively. The normal tissue types included adipose, adrenal gland, bladder, blood, blood vessel, bone marrow, brain, breast, cervix uteri, colon, esophagus, fallopian tube, heart, kidney, liver, lung, muscle, nerve, ovary, pancreas, pituitary, prostate, salivary gland, skin, small intestine, spleen, stomach, testis, thyroid, uterus, and vagina. Single-cell RNA-seq (scRNA-seq) data for LIHC were collected from the China National Genebank Database (CNGBdb, https://db.cngb.org/search/project/CNP0000650); accession code: CSE0000008).

### Correlation analysis

Based on prior reports in the literature, 106 AMPK substrates were extracted [11]. The expression correlation between AMPK subunits and AMPK substrates in 31 normal tissues was determined by Pearson correlation analysis (Correlation coefficients > 0.2).

### Differential expression analysis of AMPK subunits

To explore differences in the expression of AMPK subunits between tumor samples and their matched normal tissue controls in pan-cancer, we used the gene set cancer analysis (GSCA) online tool (http://bioinfo.life.hust.edu.cn/GSCA/). A false discovery rate-adjusted *p-*value less than 0.05 indicated a significant difference.

### Survival and immune infiltration analyses

Based on the median *PRKAA2* level, patients were divided into high- and low-expression subgroups. The R packages “*survival*” and “*survminer*” were employed for Kaplan–Meier survival analyses. The abundance of each infiltrating immune cell type was analyzed using a single-sample gene set enrichment analysis. Wilcoxon signed-rank test was performed and *p* less than 0.05 implied a significant difference.

### Single-cell RNA-seq data processing

The scRNA-seq matrices were collected for more than 300 transcripts/cell and more than 3 cells/gene condition. The NormalizeData function from the R package “*Seurat*” was applied to normalize scRNA-seq data. The clustering analysis was conducted based on the integrated joint embedding generated by the Harmony algorithm. The top 15 principal components and the top 2000 variable genes were selected for subsequent analysis. Cell clusters were detected using the FindClusters function in Seurat (resolution = 0.6), and cell clustering results were visualized by uniform manifold approximation and projection or t-distributed stochastic neighbor embedding (t-SNE) analysis.

### Cell–cell communication analysis

We performed cell communication analysis using CellphoneDB [20]. Average expression levels were calculated based on the annotated ligand-receptor pairs attained from the STRING database. The ligand-receptor pairs with *p* < 0.05 values were identified, and the interactions between two cell types using these identified pairs were analyzed. Cytokines play a critical role in cell communication; thus, cytokine signaling, based on the transcriptomic profiles, was determined using CytoSig.

### Pseudotime analysis

To detect the association between *PRKAA2* expression and T-cell evolution during tumor progression in single cells, a Pseudotime analysis was performed using R packages “*monocle2*” [21]*.* The monocle subject was built by applying the function “newCellDataSet”. Trajectory analysis was performed based on the differentially expressed genes determined by the R package “*Seurat*”. The “reduceDimension” function was used for dimensionality reduction, and cells were placed on Pseudotime trajectories using the “orderCells” functions.

### Metabolic pathway analysis

Single-cell metabolic activity was evaluated using the R package “scMetabolism” based on a previously reported method [22]. Differences in the metabolic pathway scores between subgroups with high or low *PRKAA2* expression were analyzed using Wilcoxon signed-rank test, and *p* less than 0.05 implied a significant difference.

### Functional enrichment analysis

Gene Ontology (GO) and Kyoto Encyclopedia of Genes and Genomes (KEGG) analyses were conducted using Metascape. Genes enriched more than 3-fold and *p* less than 0.05 were considered significantly different.

### Cell culture and gene expression assays

The human LIHC cell line, HepG2, was acquired from the American Type Cell Culture Collection. Cells were grown in Dulbecco Modified Eagle Medium (Gibco, Thermo Fisher Scientific, Waltham, MA, USA) containing 10% fetal bovine serum (FBS, BI) at 37 °C and 5% CO_2_. *PRKAA2* knockout cells were generated by transfecting cells with the lentiviral-based short hairpin RNA (shRNA) vector pGPU6/GFP/Neo (Genechem, Shanghai, China). The shRNA sequences were as follows: shRNA-1: GTGGCTTATCATCTTATCATT and shRNA-2: GTCATCCTCATATTATCAAAC. *PRKAA2* mRNA levels were measured using quantitative real-time PCR (qPCR). Total RNA was extracted using an RNA extraction reagent (Takara, 9108, Japan), and 2X Super SYBR Green qPCR Master Mix (ES Science, Guangzhou, China) was used to detect *PRKAA2* levels. The qPCR primer sequences for *PRKAA2* and *GAPDH* were as follows: *PRKAA2* forward, 5′-CGGGTGAAGATCGGACACTA-3′;*PRKAA2* reverse, 5′-TCCAACAACATCTAAACTGCGA′; *GAPDH* forward, 5′-GACCTGACCTGCCGTCTA-3′;and *GAPDH* reverse, 5′-AGGAGTGGGTGTCGCTGT-3′.

### Cell proliferation, clonogenic, migration, and invasion assays

Cells (8000/well) were seeded into 96-well plates (Servicebio, WuHan, China), and proliferation was evaluated using Cell Counting Kit-8 (Biosharp Biotechnology, Beijing, China) reagent. The optical density was read at 450 nm. For clone formation assays, cells were cultured for 14 days, fixed with 4% paraformaldehyde for 15 min, and stained with 0.1% crystal violet for 15 min (Meilunbio, Dalian, China). For scratch assays a linear wound was created in a monolayer of serum-starved cells using a 10-μL pipette tip, and cell coverage across this line was determined. For Transwell migration assays, cells were seeded into the upper chamber (8 μm; BIOFIL, Guangzhou, China) and incubated with serum-free medium. The lower chamber contained medium with 10% FBS. After 24 hours, cells were fixed with 4% paraformaldehyde and stained with 0.1% crystal violet.

### Statistical analysis

Continuous variables were compared using Student’s T-test (parametric) analyzed with GraphPad Prism 8 software or Wilcoxon rank sum test (nonparametric) analyzed with R software package. In vitro experiments were repeated three times, and all results are presented as means ± standard deviation. A *p-*value less than 0.05 indicates a significant difference.

## Results

### Expression patterns and physiological functions of AMPK in human tissues

**Fig. 1:**
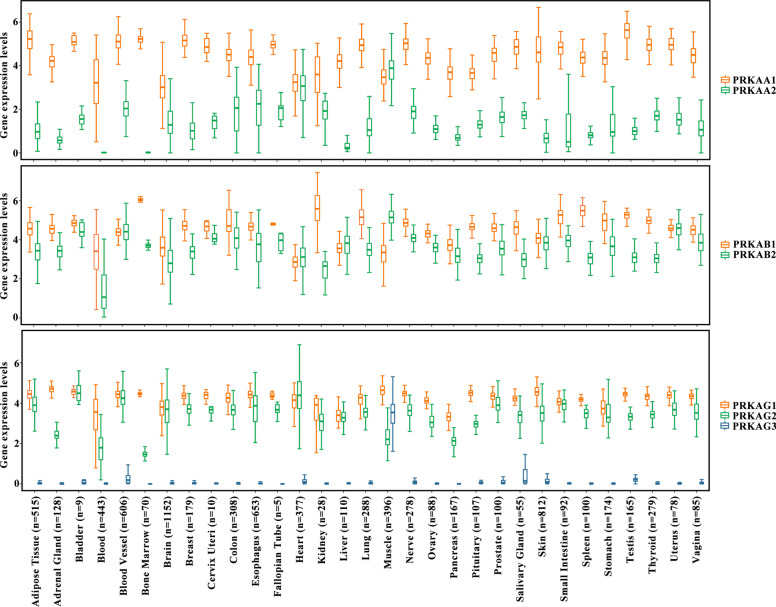
The expression levels of AMPK subunits in 31 tissue types. AMPK subunits were grouped based on the subunit types. The normal tissue types included adipose, adrenal gland, bladder, blood, blood vessel, bone marrow, brain, breast, cervix uteri, colon, esophagus, fallopian tube, heart, kidney, liver, lung, muscle, nerve, ovary, pancreas, pituitary, prostate, salivary gland, skin, small intestine, spleen, stomach, testis, thyroid, uterus, and vagina, and the data came from the Genotype-Tissue Expression database

The expression levels of AMPK subunits, including AMPKα (α1 and α2), AMPKβ (β1 and β2), and AMPKγ (γ1, γ2, and γ3), in different human tissues were assessed using GTEx bulk RNA-seq data. *PRKAA1*, *PRKAB1*, and *PRKAG1* were the main subunits expressed in almost all tissue types. *PRKAA2*, *PRKAB2*, and *PRKAG3* were the predominant subunits expressed in muscle tissues and *PRKAG2* was the major subunit expressed in heart tissues (Fig. 1). Thus, the expression of AMPK subunits was tissue-specific.

**Fig. 2:**
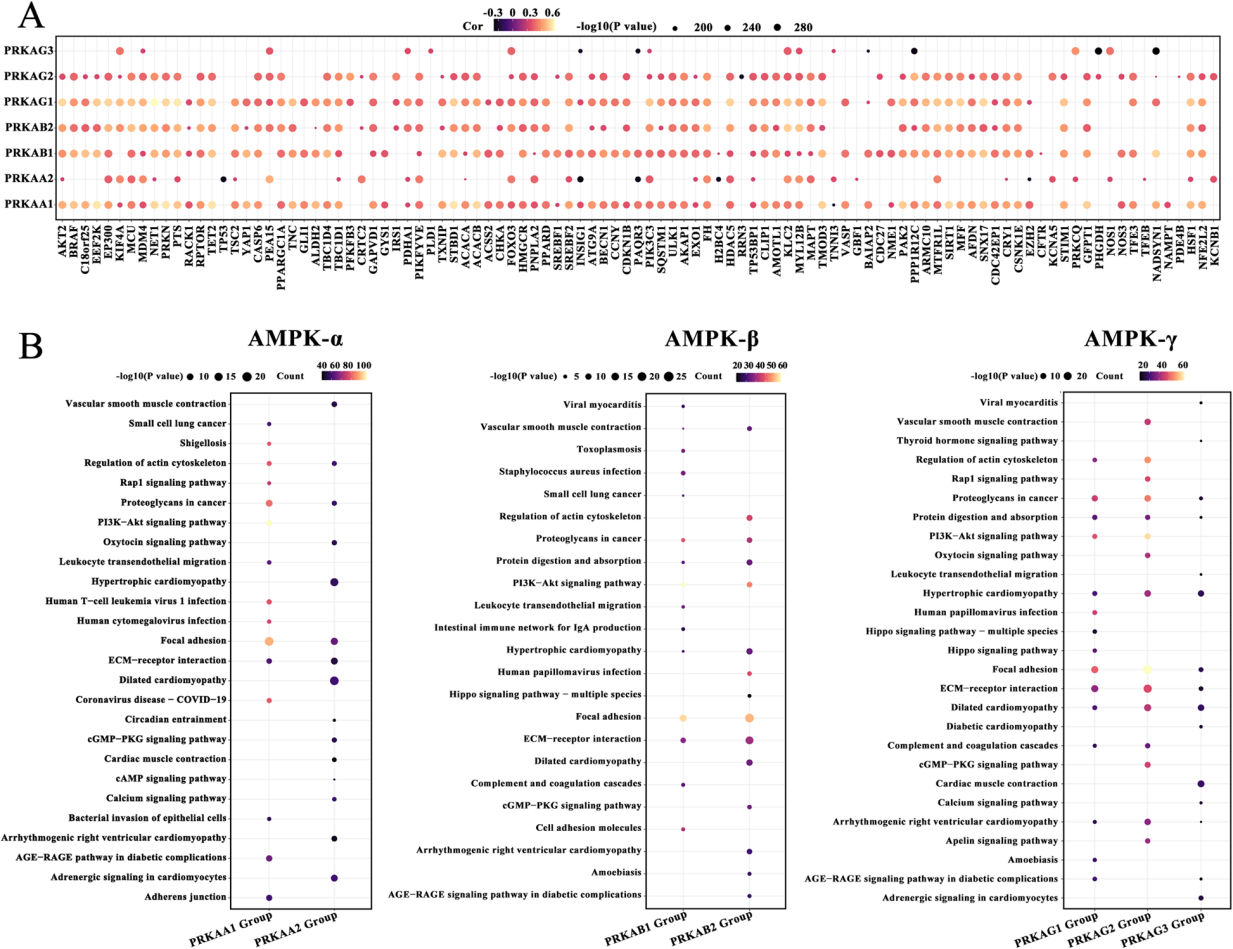
Substrate specificity of AMPK. **A** Association between the expression of AMPK subunits and AMPK substrates (Pearson correlation test; correlation coefficients > 0.2). **B** Kyoto Encyclopedia of Genes and Genomes analysis of signaling pathway enrichment in different AMPK subunit groups. The circle color represents the number of enriched pathways, and the circle size indicates the *p*-value

AMPK exerts biological functions by activating distinct substrates. Thus, gene expression correlations between different subunits of AMPK and AMPK substrates were assessed. As shown in Fig. 2A, different AMPK subunits significantly correlated with distinct substrates. *PRKAA1* expression correlated with BRAF, C18orf25, EEF2K, and EP300 expression. *PRKAA2* expression correlated with CRTC2, TNNI3, KCNA5, and TFEB expression. These results indicate that AMPK subunits may be characterized by substrate specificity. Differences in the enriched pathways for different AMPK subunits were determined using KEGG analysis. Diverse AMPK isoforms participated in different regulatory programs (Fig. 2B). These results indicate that the substrate specificity of AMPK isoforms may lead to functional differences between AMPK isoforms.

### Relationship between *PRKAA2* expression, LIHC prognosis, and immune cell infiltration

**Fig. 3:**
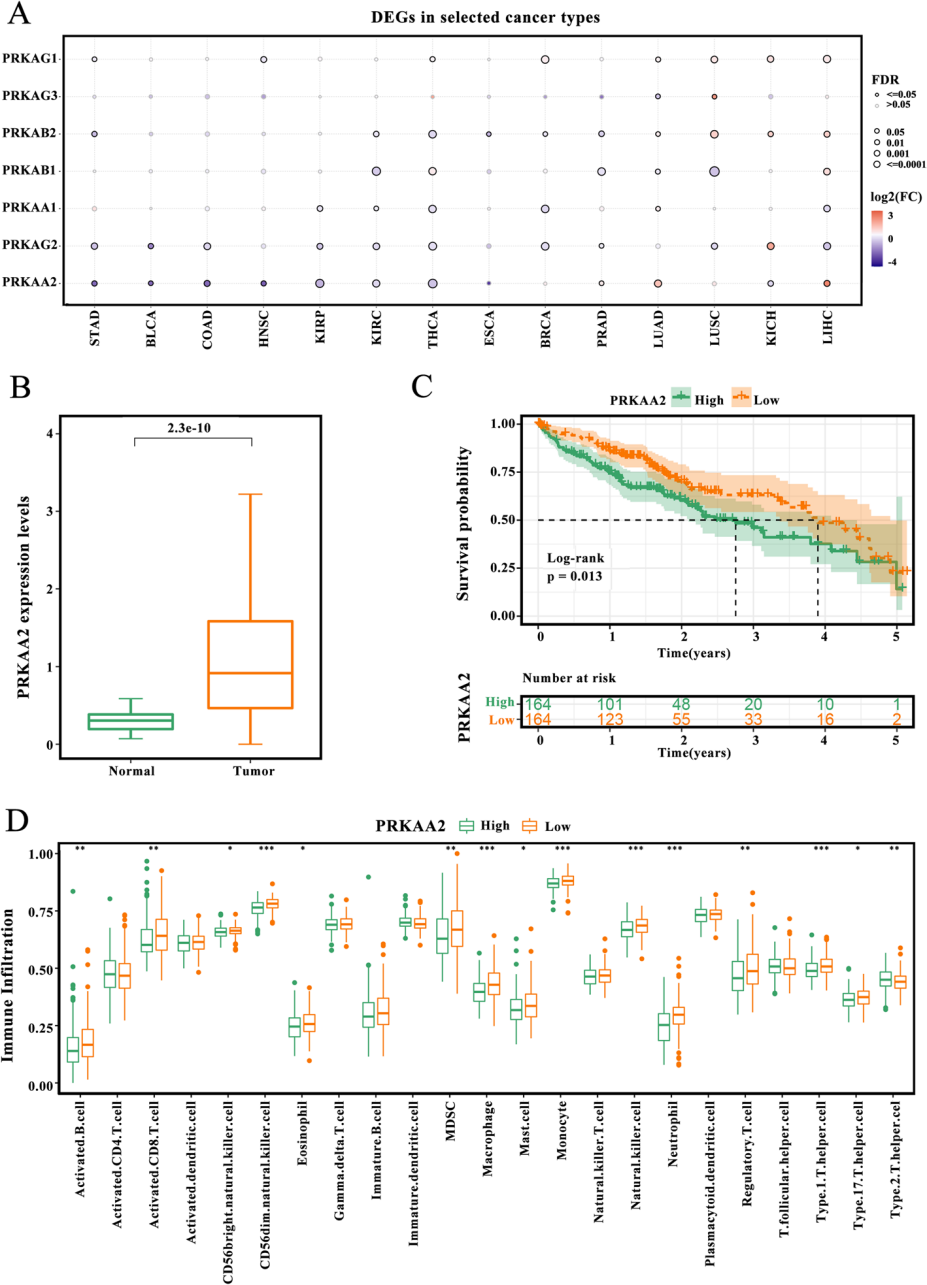
*PRKAA2* expression correlates with prognosis and immune cell infiltration. **A** AMPK subunit expression levels in tumor tissues and matched adjacent normal tissues in 14 tumor types were evaluated using the gene set cancer analysis (GSCA) database. An adjusted *p*-value < 0.05 indicates a significant difference. **B** The expression of *PRKAA2* in tumor tissues and normal controls in the TCGA-LIHC cohort (Wilcoxon rank test; tumor samples: *n* = 368, normal tissues: *n* = 50). **C** Differences in survival probability between patients with high *PRKAA2* expression and patients with low *PRKAA2* expression in the TCGA-LIHC cohort (log-rank test). **D** The difference in tumor-infiltrating immune cell scores between patients with high *PRKAA2* expression and patients with low *PRKAA2* expression in the TCGA-LIHC cohort (*n* = 368; Wilcoxon test; *P < 0.05; **P < 0.01; ***P < 0.001)

The expression levels of AMPK subunits in tumor tissue and matched adjacent normal tissue were compared using the GSCA database. Details of datasets were shown in Table S1. P*RKAA2* was differentially expressed in most tumor types (Fig. 3A). Of note, *PRKAA2* was significantly upregulated in LIHC, and this upregulation was in the TCGA-LIHC cohort (Fig. 3B). Subsequent Kaplan-Meier survival analyses indicated that high *PRKAA2* expression was linked to unfavorable prognosis in patients with LIHC (Fig. 3C). The TME status in response to different *PRKAA2* expression patterns was determined by calculating the infiltration abundances of 23 immune cell types. Patients with low *PRKAA2* expression displayed a significantly higher degree of immune cell infiltration, indicative of an immune hot phenotype, compared with patients with high *PRKAA2* expression, which exhibited an immune cold phenotype lacking immune cell infiltration (Fig. 3D).

### High *PRKAA2* expression correlated with LIHC progression in malignant cells

**Fig. 4:**
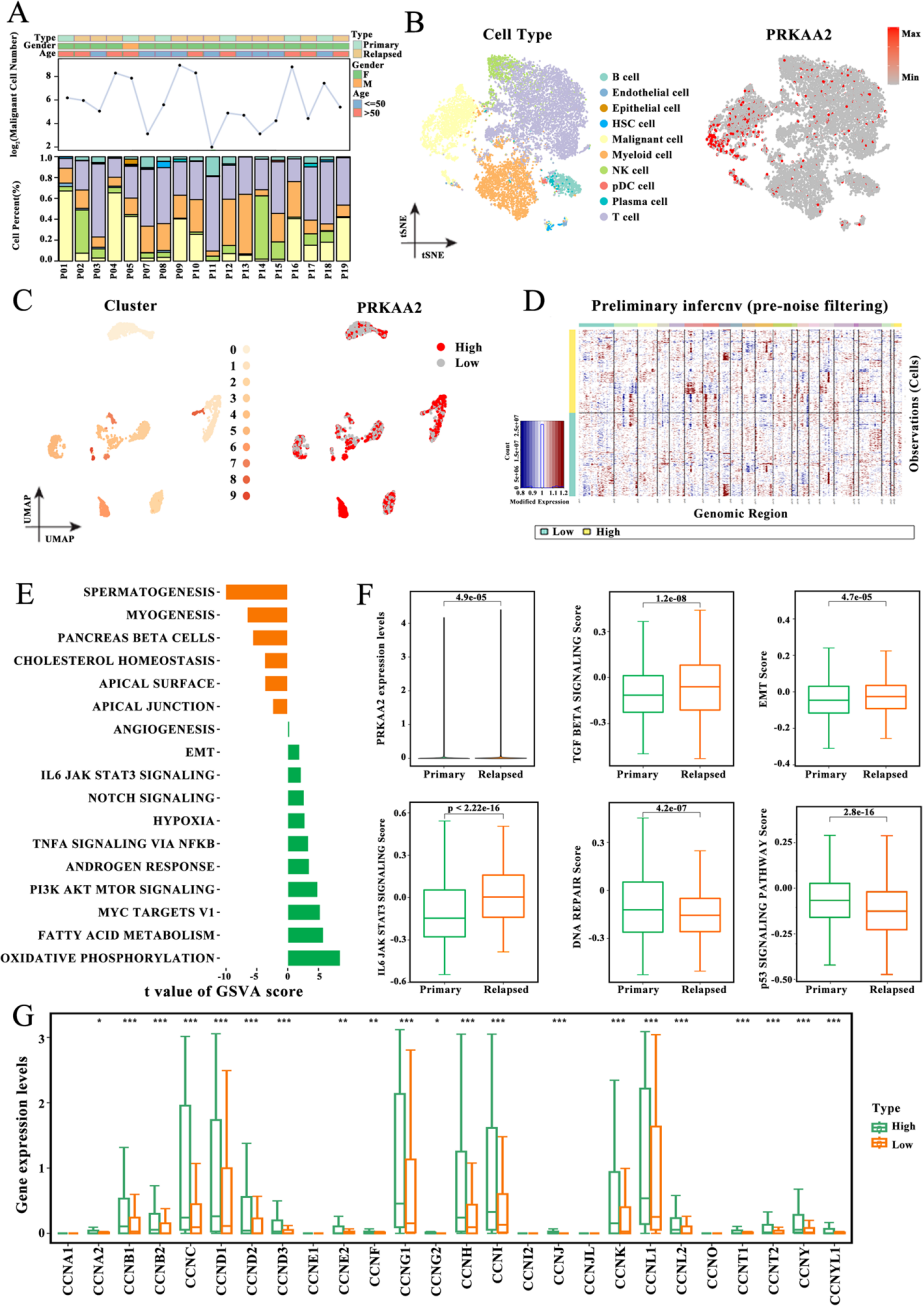
*PRKAA2* expression in malignant cells is associated with liver hepatocellular carcinoma (LIHC) development. **A** Clinical and molecular features of 18 LIHC patients from the Chinese National Genebank Database (CSE0000008) and the proportion of cell types in each patient. **B** T-distributed stochastic neighbor embedding plot of 14,236 cells colored based on cell types or *PRKAA2* expression. **C** Uniform manifold approximation and projection plot of 2730 malignant cells colored based on cell subgroups or *PRKAA2* expression. **D** Heatmap of copy number profile genes in malignant cells with high *PRKAA2* expression and low *PRKAA2* expression. **E** The enriched pathways in malignant cells with high *PRKAA2* expression and low *PRKAA2* expression using gene set variation analysis (Bayesian t-test). **F** Differences in *PRKAA2* expression and pathway scores between primary tumors and relapsed tumors (Wilcoxon rank test). **G** Differences in the expression of cyclin genes between malignant cells with high *PRKAA2* expression and low *PRKAA2* expression (Wilcoxon rank test; *P < 0.05, **P < 0.01, ***P < 0.001)

The scRNA-seq dataset for LIHC was obtained from the CNGBdb (CSE0000008). The dataset included 12 primary tumors and 6 relapsed tumors (Fig. 4A). Ten cell subgroups were identified using the analysis of t-SNE clustering of single-cell samples. Based on the gene expression of cell-type specific markers, cell subgroups were annotated as known cell lineages, including immune cells (B cells, myeloid cells, NK cells, pDC, plasma cells, and T cells), malignant cells, hematopoietic stem cells, endothelial cells, and epithelial cells (Fig. 4B). *PRKAA2* was predominantly expressed in malignant cells. Malignant cells were extracted and clustered. As shown in Fig. 4C, a total of 10 subgroups were obtained, and cells with high *PRKAA2* expression clustered mainly in subgroups 1, 6, and 7, indicating heterogeneity in *PRKAA2* expression. Malignant cells in the high *PRKAA2* expression group exhibited a more malignant phenotype than cells in the low *PRKAA2* expression group (Fig. 4D). Gene set variation analysis (GSVA) revealed that signaling pathways associated with tumor progression, including PI3K/Akt/mTOR signaling, Notch signaling, angiogenesis signaling, and epithelial–mesenchymal transition (EMT), were significantly enriched in malignant cells with high *PRKAA2* expression (Fig. 4E). *PRKAA2* expression was higher in malignant cells from relapsed tumors compared with *PRKAA2* expression in malignant cells from primary tumor samples. Genes involved in TGF-β signaling and EMT were also higher in malignant cells from relapsed tumors compared with malignant cells from primary tumor samples (Fig. 4F). Conversely, DNA repair and p53 signaling pathways were more pronounced in primary tumor samples. Finally, cyclins were upregulated in malignant cells with high *PRKAA2* expression, suggesting that these cells are in the activated cell cycle state (Fig. 4G). Altogether, these findings reveal that *PRKAA2* may contribute to LIHC development.

### *PRKAA2* contributes to immune escape in LIHC

**Fig. 5:**
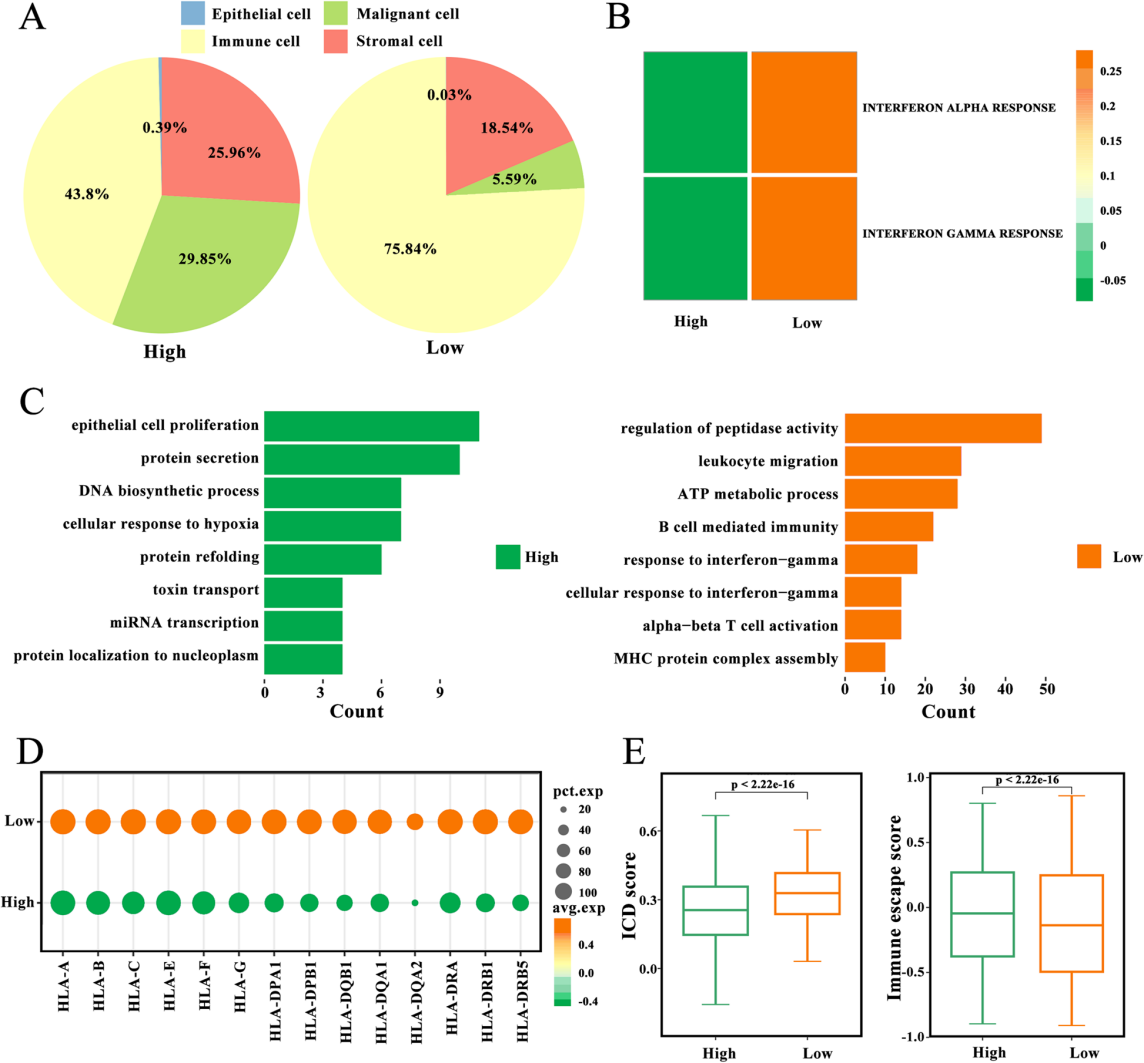
*PRKAA2* is associated with the immune escape of tumor cells. **A** The proportion of epithelial cells, immune cells, malignant cells, and stromal cells in tumors with high *PRKAA2* expression and low *PRKAA2* expression. **B** Differences in interferon-alpha (IFN-α) and interferon-gamma (IFN-γ) pathway scores between malignant cells from high and low *PRKAA2* expression patient subgroups. Gene set variation analysis was performed. **C** Analysis of Gene Ontology enrichment of differentially expressed genes between high and low *PRKAA2* expressing malignant cells. **D** The levels of human leukocyte antigen molecules in high and low *PRKAA2* expressing malignant cells. **E** Differences in the enriched immunogenic cell death pathways scores and immune escape between two different scoring subgroups (Wilcoxon rank test)

To evaluate the relationship between *PRKAA2* and the LIHC immune microenvironment, patients (*n* = 18) were divided into high and low *PRKAA2* expression groups. The proportion of immune cells was significantly lower in tumors with high *PRKAA*2 expression compared with the proportion of immune cells in the low *PRKAA2* expression group (43.8% vs. 75.84%; Fig. 5A). Single-sample GSVA to detect differentially expressed genes between the two subgroups revealed that the IFN-γ response pathways were enriched in the malignant cells of tumors with low *PRKAA2* expression (Fig. 5B). This finding was confirmed using GO enrichment analysis (Fig. 5C). Notably, GO analysis also revealed that the MHC protein complex assembly signaling pathway was significantly upregulated in the low *PRKAA2* expression subgroup. The interferon-gamma (IFN-γ) signaling pathway plays a key role in regulating MHC-I expression. Thus, the levels of MHC-I genes were assessed in the two subgroups. Malignant cells with high *PRKAA2* expression exhibited low levels of MHC-I gene expression (Fig. 5D), indicative of weak immunogenicity. In agreement with this finding, the high-expression group also had a significantly low immunogenic cell death pathway score. The functional consequence of high *PRKAA2* expression was increased immune escape (Fig. 5E).

### *PRKAA2* associated with dynamic changes in T cells during LIHC progression

**Fig. 6:**
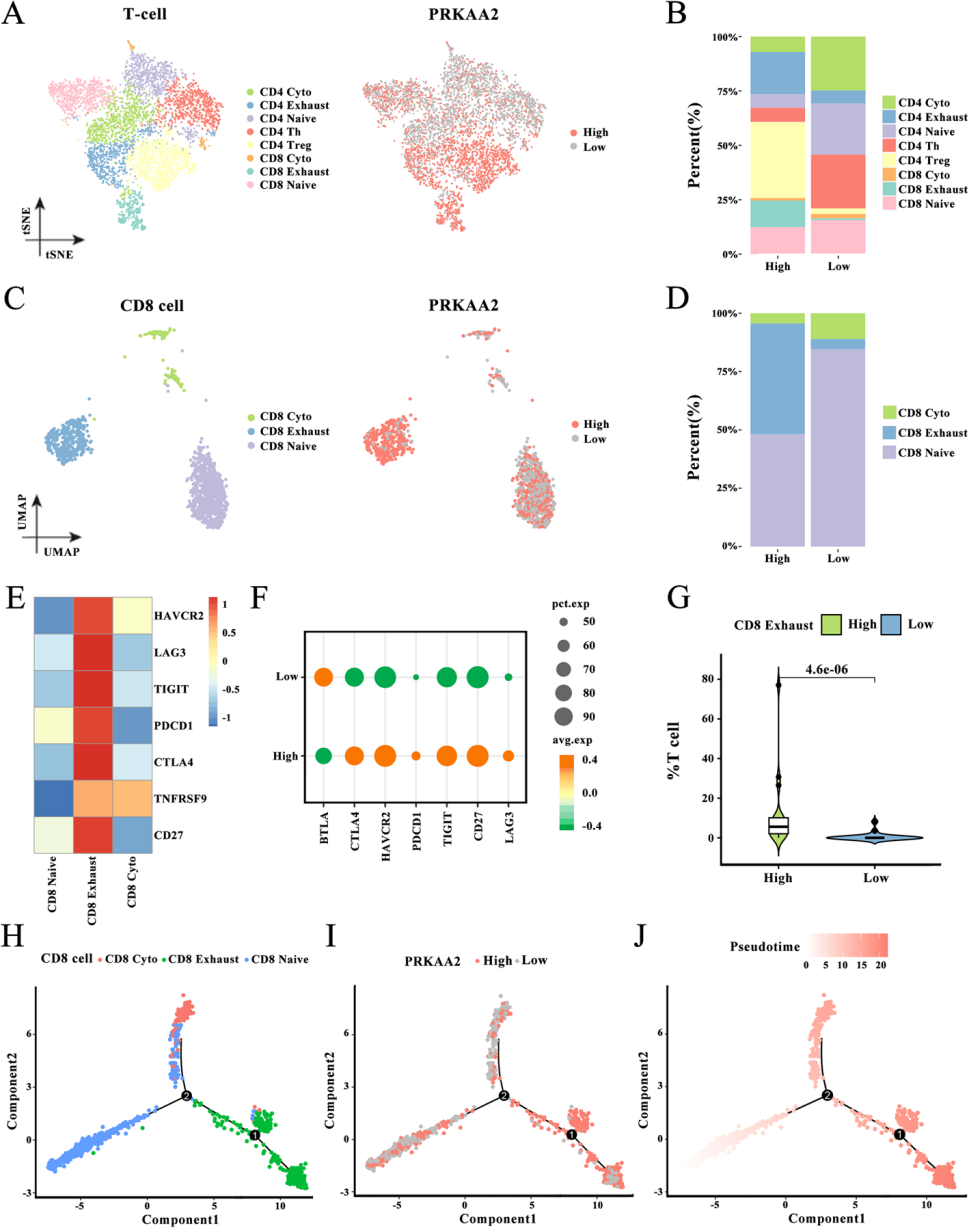
*PRKAA2* expression is associated with dynamic change in CD8+ T cells during tumor progression. (**A**) T-distributed stochastic neighbor embedding plot of 6179 T cells colored based on cell types or *PRKAA2* expression. **B** The proportions of eight types of T cells with high or low *PRKAA2* expression levels. **C** Uniform manifold approximation and projection plot of 1366 CD8+ T cells colored based on cell types or *PRKAA2* expression levels. **D** The proportions of CD8+ cyto, CD8+ exhausted, and CD8+ native T cells with high or low *PRKAA2* expression. (E-F) The levels of immune checkpoint proteins, including CTLA4, HAVCR2, PDCD1, TIGIT, CD27, and LAG3 among the CD8+ cyto, CD8+ exhausted, and CD8+ native T cells **E** and in T cells expressing high or low levels of *PRKAA2* **F**. **G** The proportion of CD8+ exhausted T cells expressing high or low levels of *PRKAA2* (Wilcoxon rank test). **H-J** Differentiation trajectory of CD8+ T cells, colored for cell types **H**, *PRKAA2* expression **I**, and pseudotime **J**

To explore the relationship between *PRKAA2* and the evolution of T cells, we first identified 6179 T cells. All T cells were clustered into eight subgroups and annotated as CD4^+^ T cells (cytotoxic CD4^+^ T cells, exhausted CD4^+^ T cells, native CD4^+^ T cells, CD4^+^ T helper (Th) cells, and CD4^+^ T regulatory (Treg) cells) and CD8^+^ T cells (cytotoxic CD8^+^ T cells, exhausted CD8^+^ T cells, and native CD8^+^ T cells) (Fig. 6A). T cells with high *PRKAA2* expression were primarily enriched in exhausted T cells and CD4^+^ Treg cells. These results were confirmed by analyzing the composition and proportion of T-cell types in high and low *PRKAA2* expression subgroups (Fig. 6B). Compared with the other CD8+ T-cell types, exhausted CD8+ T cells expressed higher levels of *PRKAA2* (Fig. 6C-D). In addition, the expression levels of multiple immune checkpoint proteins, including CTLA4, HAVCR2, PDCD1, TIGIT, CD27, and LAG3, were higher in the exhausted CD8+ T-cell subgroup with high *PRKAA2* expression (Fig. 6E-G), implying that the functional exhaustion of CD8+ T cells may be a potential mechanism for *PRKAA2*-mediated cancer cell immune evasion. Immune cells in the tumor microenvironment evolve with the progression of the tumor, and this dynamic process can be described by the monocle algorithm. Pseudotime and trajectory analyses showed that CD8+ T cells tended to be exhausted during tumor progression, which was associated with high expression of *PRKAA2* (Fig. 6H-J). Thus, *PRKAA2* may promote tumor progression by contributing to CD8+ T-cell exhaustion.

**Fig. 7:**
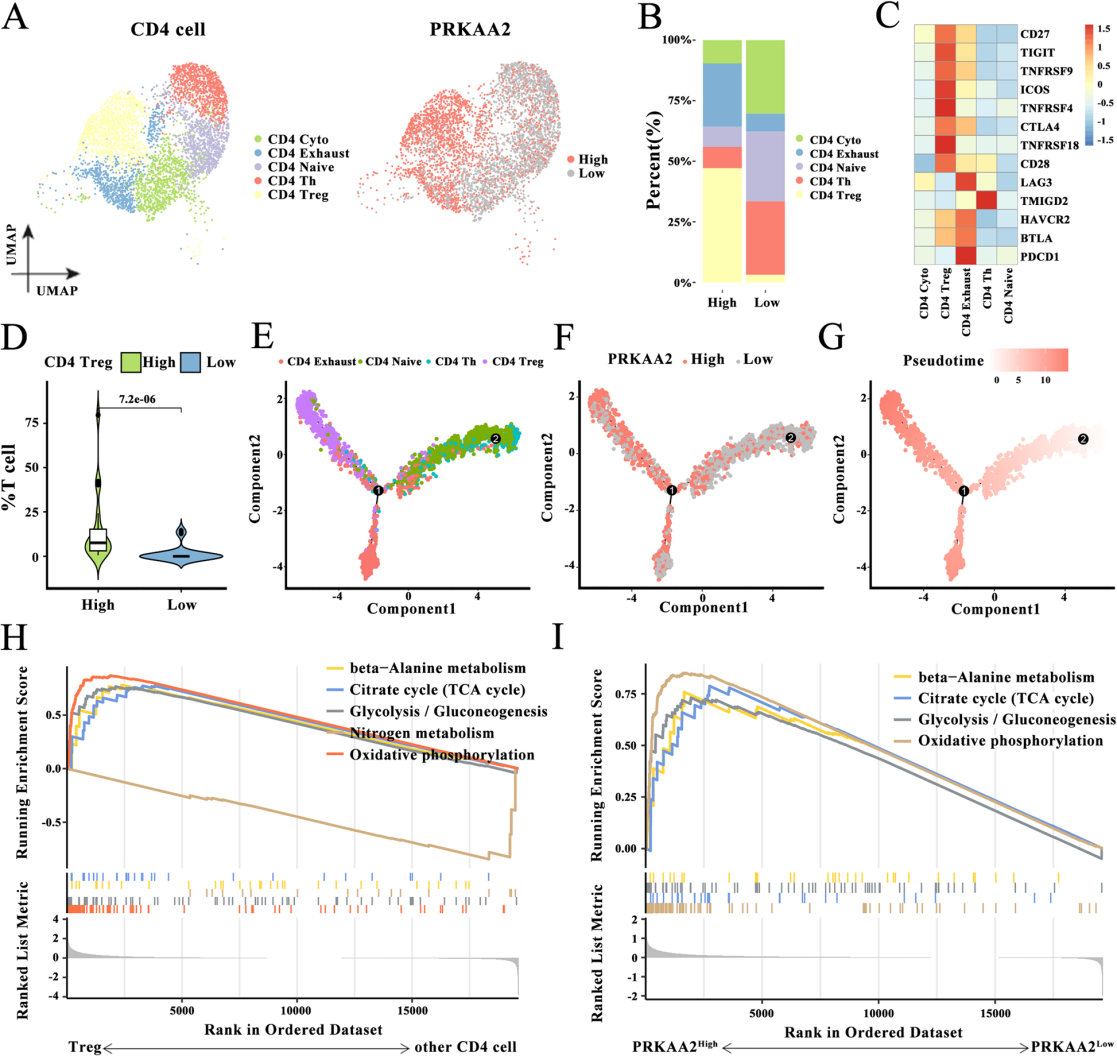
*PRKAA2* is associated with dynamic change of CD4+ T cells during tumor progression. **A** Uniform manifold approximation and projection plot of 4813 CD4+ T cells colored based on cell types or *PRKAA2* expression. **B** The proportion of five types of CD4+ T cells expressing high or low levels of *PRKAA2*. **C** The levels of immune checkpoint proteins, including CD27, TIGIT, TNFRSF9, ICOS, TNFRSF4, CTLA4, TNFRSF18, and CD28 in the five types of CD4+ T cells. **D** The proportion of CD4+ Treg cells expressing high or low levels of *PRKAA2* (Wilcoxon rank test). (E-G) Differentiation trajectory of CD4+ T cells colored for cell types **E**, *PRKAA2* expression **F**, and pseudotime **G**. (H-I) The significantly enriched metabolic pathways in Treg cells **H** and T cells with high *PRKAA2* expression **I** were identified by gene set enrichment analysis

CD4+ Treg cells, which are an important component of CD4+ T-cell types, are mainly responsible for maintaining immunological tolerance and homeostasis. High *PRKAA2* expression in CD4+ Treg cells significantly enhanced CD27, TIGIT, TNFRSF9, ICOS, TNFRSF4, CTLA4, TNFRSF18, and CD28 expression levels (Fig. 7A-D). This suggests that CD4+ Treg cells mediate the promotion of tumor immune escape by *PRKAA2*. Differentiation trajectory analysis of CD4+ T cells revealed that *PRKAA2* may be involved in the formation of CD4+ Treg cells (Fig. 7E-G). Increased metabolism of Treg cells is a key factor in maintaining their immunosuppressive effects. Thus, the enrichment of metabolic-related signaling pathways in Treg cells and other T-cell types was examined. The metabolic pathways enriched in Treg cells included beta-alanine metabolism, citrate cycle (TCA cycle), glycolysis/gluconeogenesis, and oxidative phosphorylation (Fig. 7H). These signaling pathways were also enriched in T cells with high *PRKAA2* expression (Fig. 7I). Taken together, our findings suggest that *PRKAA2* may contribute to CD8+ T-cell exhaustion and the formation of CD4+ Treg cells to facilitate immune escape.

### Reshaping intercellular interactions by *PRKAA2* in the TME

**Fig. 8:**
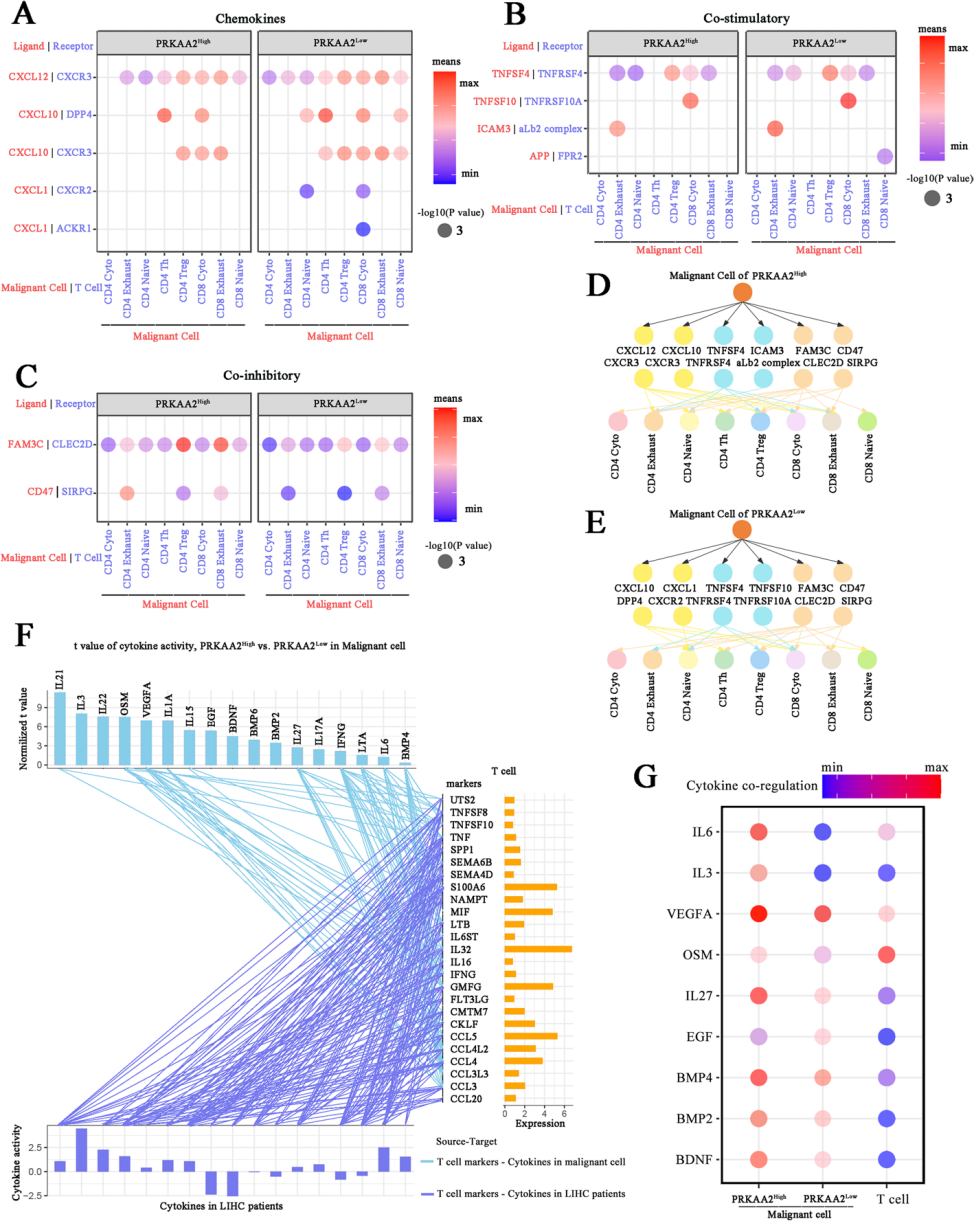
Cell-cell communication analysis. (A-C) Malignant cell-T-cell interactions through chemokines (**A**), costimulatory molecules (**B**), and coinhibitory molecules (**C**). Circle size and color indicate the *P* values and expression levels of ligand-receptor molecules, respectively. (D-E) Ligand-receptor connections between malignant cells and T-cell subtypes with high (**D**) or low (**E**) *PRKAA2* expression. (**F**) The activities of cytokine pathways in *PRKAA2* high-expression malignant cells or liver hepatocellular carcinoma (LIHC) patients positively linked to T-cell markers (*r* > 0.2 and *P* < 0.05). (**G**) Signaling activities of cytokines in malignant cells and T cells with high and low *PRKAA2* expression

The interaction between malignant cells and T cells within the TME is crucial for tumor progression. Therefore, a cell-cell communication network was built to explore the role of *PRKAA2* in cell communication. The expression levels of chemokines from malignant cells, including CXCL12, CXCL10, and CXCL1, were significantly changed by *PRKAA2* (Fig. 8A). In addition, high *PRKAA2* expression suppressed costimulatory molecules, including TNFSF4, TNFSF10, ICAM3, and APP, generated by malignant cells (Fig. 8B). However, coinhibitory signals were enhanced in malignant cells with high *PRKAA2* expression (Fig. 8C). Of note, synergistic interaction between T cells and malignant cells with high *PRKAA2* expression activated Treg cells and promoted T-cell exhaustion (Fig. 8D). Conversely, induction of multiple ligand-receptors pairs, including CXCL10/DPP4, CXCL1/CXCR2, TNFSF4/TNFRSF4, and FAM3C/CLEC2D, reinforced the accumulation of T cells in response to low *PRKAA2* expression (Fig. 8E).

Cytokines regulate cell-cell communications within the immune system. Thus, the relationship between *PRKAA2* and cytokine signaling at the single-cell level was investigated. Seventeen cytokine-related pathways were activated in malignant cells with high *PKRAA2* expression; 10 of these pathways were significantly and positively associated with marker genes from T cells (Fig. 8F). Differences in the activation of cytokine signaling pathways, such as IL6 and IL3, between high and low *PRKAA2* expression subgroups contributed to the distinct communication behaviors in T cells and malignant cells (Fig. 8G). Consequently, the functional role of *PRKAA2* in reshaping cell-cell interactions and cytokine signaling activity in the TME suggests that *PRKAA2* is a main regulator of T-cell exhaustion in LIHC.

### *PRKAA2*-mediated metabolic reprogramming in malignant cells

**Fig. 9:**
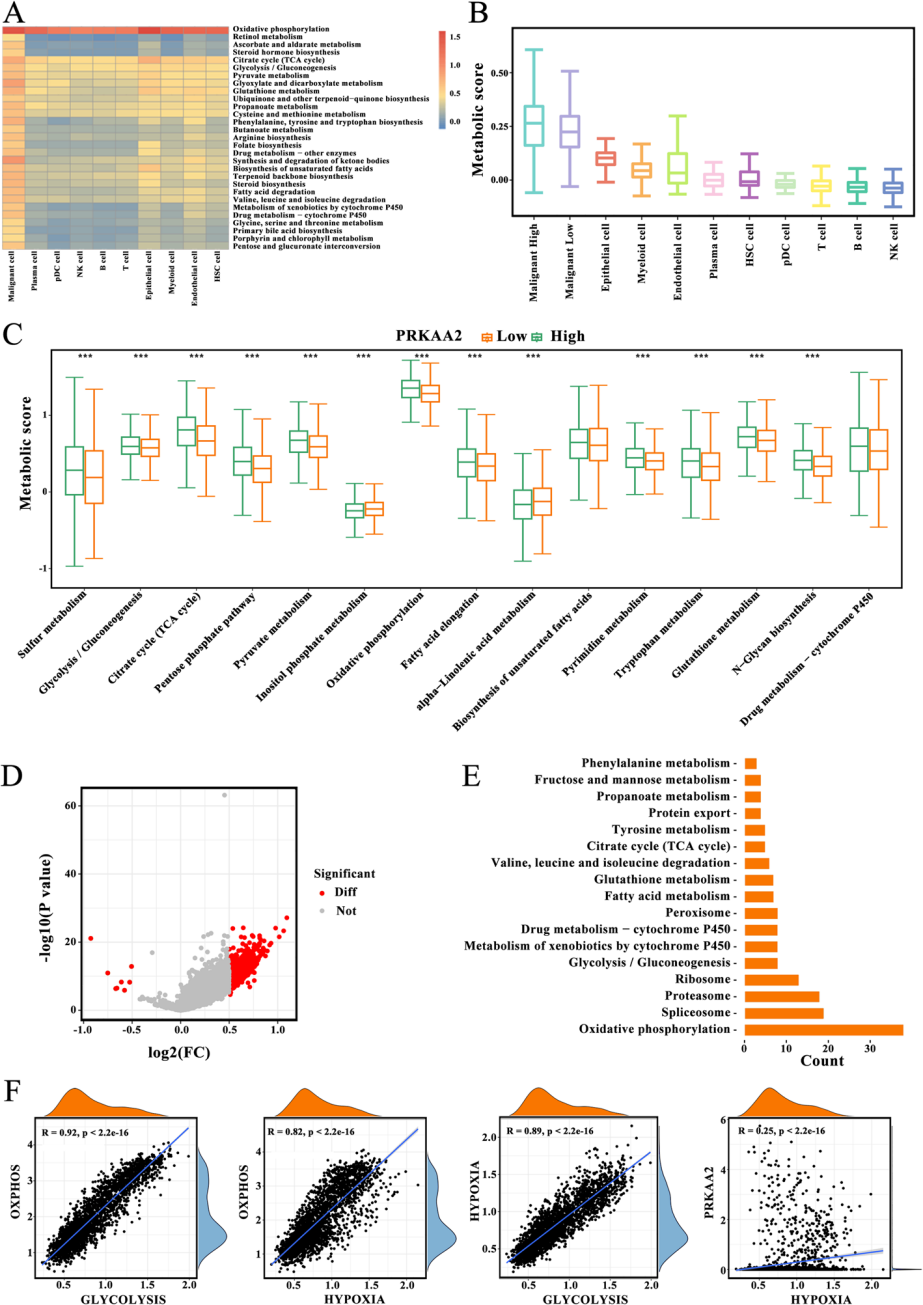
Metabolic landscape for liver hepatocellular carcinoma with different *PRKAA2* expression patterns. **A** The activities of metabolic signaling pathways in each cell type, including malignant cells, plasma cells, pDC, NK cells, B cells, T cells, epithelial cells, myeloid cells, endothelial cells, and hematopoietic stem cells (Wilcoxon rank test; *P* < 0.05). **B** Overall metabolic pathway abundance in plasma cells, pDC, NK cells, B cells, T cells, epithelial cells, myeloid cells, and endothelial cells, and in malignant cells with high and low *PRKAA2* expression. **C** Differences in the metabolic scores in malignant cells with high or low *PRKAA2* expression levels (Wilcoxon rank test; ***P < 0.001). **D** Differentially expressed genes in malignant cells expressing high or low levels of *PRKAA2* (Wilcoxon rank test; ***P < 0.001). **E** Gene Ontology analysis of differentially expressed genes. **F** Comparison of glycolysis, oxidative phosphorylation, and response to hypoxia in malignant cells (Spearman’s rank correlation)

The role of *PRKAA2* in the metabolic reprogramming of malignant cells was investigated. Compared to other cell types, metabolic pathways, including glycolysis/gluconeogenesis and TCA cycle, were upregulated in malignant cells (Fig. 9A). Overall metabolic pathway abundance was significantly higher in malignant cells with high *PRKAA2* expression compared with malignant cells with low *PRKAA2* expression (Fig. 9B). Differences in each metabolic pathway activity between high- and low-expression subgroups are shown in Fig. 9C. Differentially expressed genes in the two subgroups were extracted (Fig. 9D) and a KEGG analysis was conducted. The analysis shows that genes were mainly enriched in metabolism-related signaling pathways such as oxidative phosphorylation, glycolysis/gluconeogenesis, and TCA; oxidative phosphorylation was the most enriched pathway (Fig. 9E). Oxidative phosphorylation was closely related to glycolysis and hypoxia, and glycolysis was substantially linked to the response to hypoxia (Fig. 9F). We also observed that *PRKAA2* was significantly associated with hypoxia. Thus, associations between oxidative phosphorylation and glycolysis and between oxidative phosphorylation and hypoxia indicate that coupling between aerobic respiration and hypoxia-associated pathways may be a feature of malignant cells in the TME in response to high *PRKAA2* expression.

### The role of *PRKAA2* in the development of LIHC

**Fig. 10:**
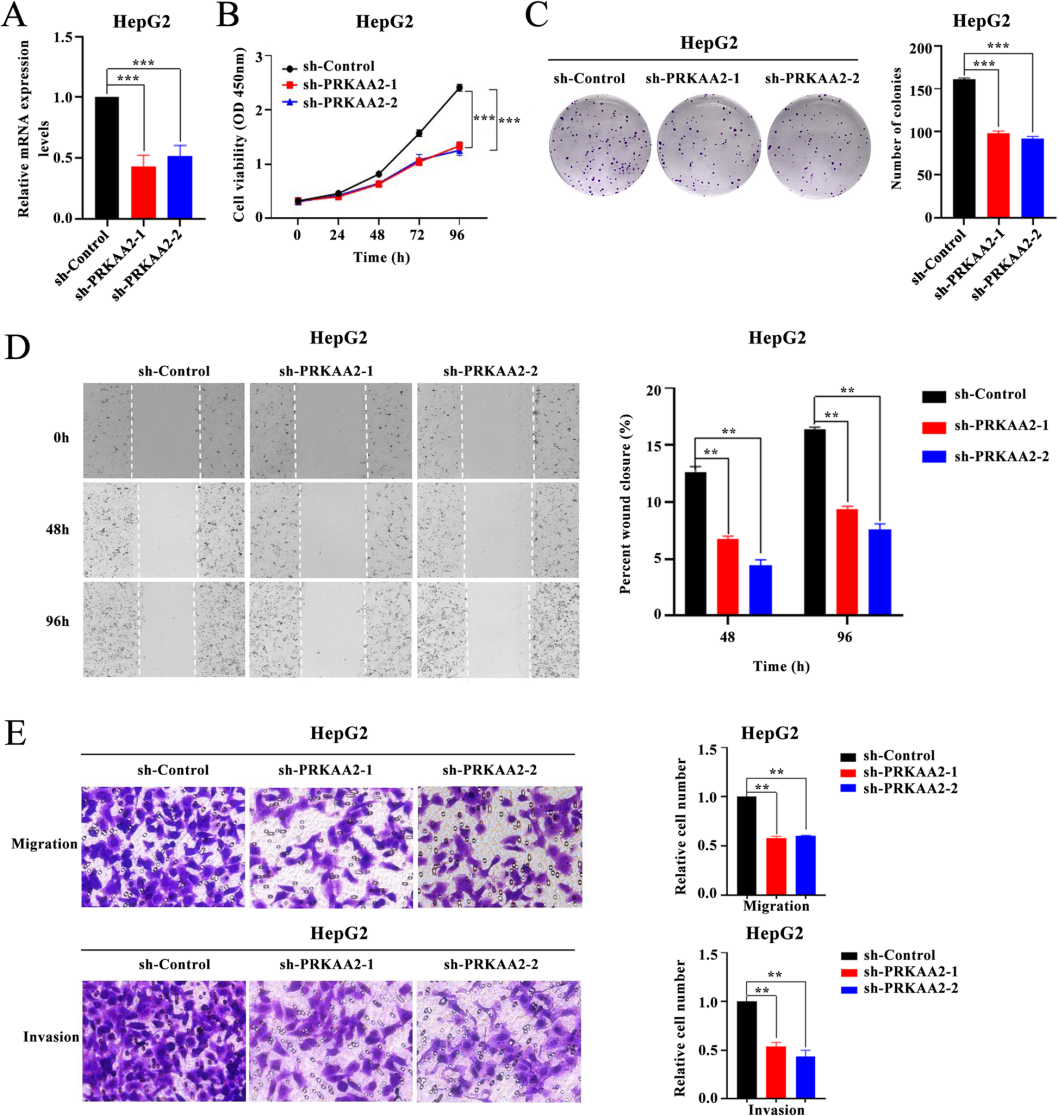
Functional role of *PRKAA2* in liver hepatocellular carcinoma. **A** *PRKAA2* expression was depleted with shRNAs in HepG2 cells. **B-E** *PRKAA2* knockdown significantly suppressed the proliferation (**B**), colony formation (**C**), scratch healing (**D**), and migration and invasion (**E**) of HepG2 cells. (Student’s t-test; *P < 0.05; **P < 0.01; ***P < 0.001)

To explore the functional role of *PRKAA2* in LIHC progression, we knocked *PRKAA2* down in LIHC HepG2 cells using shRNAs (Fig. 10A). Proliferation was significantly inhibited in *PRKAA2*-deficient HepG2 cells compared to control cells (sh-Control) (Fig. 10B). Clonogenic ability was also significantly decreased in response to *PRKAA2* silencing (Fig. 10C). *PRKAA2* depletion impaired the healing ability of HepG2 cells compared to sh-Control cells (Fig. 10D). Migratory and invasive behaviors of HepG2 cells were remarkably limited by *PRKAA2* depletion in Transwell assays (Fig. 10E). Thus, *PRKAA2* may be critical for LIHC progression and metastasis.

## Discussion

Individualized cancer therapy is paramount to improving the clinical outcomes of patients [23]. The current study reveals that AMPK plays pivotal roles in the progression and metastasis of multiple tumor types and is a potential therapeutic target for LIHC. AMPKα2, encoded by *PRKAA2*, functions as the catalytic core of AMPK; however, the role of AMPKα2 in the LIHC TME is unclear.

Our results show that AMPK subunits exhibited tissue-specific expression patterns and could be substrate-specific. *PRKAA2* was highly expressed in LIHC and was associated with poor prognosis. In vitro experiments show that *PRKAA2* knockdown inhibited the proliferation, migration, invasion, and metastasis of LIHC cells. Furthermore, *PRKAA2* expression was significantly associated with the tumor immune microenvironment. Patients with high *PRKAA2* expression lacked immune cell infiltration, indicative of an immune cold phenotype. Single-cell transcriptome atlas analysis showed that *PRKAA2* contributes to tumor progression. This conclusion is supported by the following evidence: 1) TGF-β signaling and EMT are enhanced in malignant cells; 2) metabolic reprogramming is induced in malignant cells; 3) CD8+ T-cell exhaustion and the formation of CD4+ Treg cells is promoted in T cells; and 4) Treg cell activation and T-cell depletion is driven by changes in the interactions between malignant cells and T cells in the TME. Taken together, our study demonstrates that the development of *PRKAA2*-based treatment strategies for LIHC holds great promise.

The tissue specificity of AMPK isoform distribution is the basis for a variety of AMPK biological functions [24]. AMPK can exert antitumor or tumor-promoting effects depending on the cellular context [25, 26]. AMPK activation leads to cell cycle arrest and inhibition of tumor growth, which contribute to the prevention of multiple cancer types, including lung, colorectal, and breast cancers [27–29]. In contrast, under conditions of oncogenic stress or hypoxia and nutrient deficiency in the TME, cancer cells exhibit an increased dependence on AMPK function to promote cancer cell survival [30, 31]. Our data show that functional differences between AMPK subunits could be mediated by tissue-specific expression patterns and the high substrate specificity of AMPK subunits. High expression of *PRKAA2*, which drives metabolic reprogramming and immune escape of tumor cells, contributes to LIHC development.

Our results demonstrate that the TME is closely linked to tumor heterogeneity and regulates antitumor immune responses. Among the immune cell types in the TME, T cells play a dominant role in immune regulation and antitumor activity [32]. Treg cells are an important component of immune homeostasis. Treg cells maintain immune self-tolerance and inhibit anticancer immunity [33], while CD8+ T cells are cytotoxic T lymphocytes that kill tumor cells [34]. CD8+ T cells may become exhausted during tumor progression. Our findings suggest that *PRKAA2* promotes immune escape of tumor cells via CD8+ T-cell depletion and Treg cell generation, eventually leading to tumor progression. Furthermore, malignant cells that express high levels of *PRKAA2* evade immune suppression via IFN-γ/JAK/STAT-mediated loss of MHC-I molecules [35, 36]. Altogether, *PRKAA2*-mediated tumor immune escape may be due to the activation of immune escape mechanisms in malignant cells and the formation of an immunosuppressive tumor microenvironment.

Metabolic reprogramming is a crucial pathway for the proliferation and metastasis of tumor cells [37]. Thus, understanding the features of malignant cell and non-malignant cell metabolism is important in developing a foundation for LIHC patient therapy. Our results show metabolic heterogeneity in LIHC, and malignant cells have significantly higher metabolic activity than non-malignant cells. Metabolic pathways, including oxidative phosphorylation, the TCA cycle, and glycolytic signaling, were enhanced in malignant cells that express high levels of *PRKAA2*, indicating that high *PRKAA2* expression enhances energy metabolism in malignant cells. Oxidative phosphorylation was remarkably upregulated in malignant cells, which is consistent with previous single-cell studies [38]. Interestingly, oxidative phosphorylation signaling significantly and positively correlated with hypoxia. Oxidative phosphorylation, as a sensor of oxygen availability, modulates the responses to hypoxia by stabilizing hypoxia-inducible factor [39, 40]. In support of this observation, highly dynamic interactions between oxidative phosphorylation and hypoxia were detected in another tumor study using scRNA-seq. Consequently, the positive association between oxidative phosphorylation and hypoxia-mediated by *PRKAA2* may be a unique signature of malignant cells within the TME. Collectively, the data suggest that *PRKAA2* plays an important role in the metabolic reprogramming of malignant cells.

Patients who were split into two groups according to the *PRKKA2* expression exhibited different tumor immune microenvironments, suggesting that these patients may respond to distinct treatment strategies. Tumors with low *PRKAA2* expression displayed an immune hot phenotype, characterized by a high abundance of tumor immune cell infiltrates. In contrast, patients with high *PRKAA2* expression exhibited the opposite immune characteristics, indicative of an immune cold phenotype. Therefore, inhibiting *PRKAA2* expression may convert poorly immunogenic (cold) tumors into highly immunogenic and invasive (hot) tumors. Blocking *PRKAA2* expression may restore antitumor immunity by enhancing the antitumor response of T cells and reshaping the tumor immunosuppressive microenvironment. The stratification of *PRKAA2* expression patterns can be used for developing personalized treatment approaches, contributing to the establishment of precision medicine for LIHC.

## Conclusions

Our study revealed that *PRKAA2* affects the metabolic reprogramming of malignant cells by coupling aerobic respiration with hypoxia-associated pathways. In addition, *PRKAA2* facilitates immune escape of tumor cells by promoting CD8+ T-cell exhaustion and the formation of CD4+ Treg cells by reshaping the interaction between malignant cells and T cells and driving dynamic changes in T cells in the TME. Patients with high *PRKAA2* expression may benefit from inhibitors of AMPK/AMPKα2 signaling. This study provides a theoretical basis for the development of *PRKAA2*-based personalized therapy strategies.

### Supplementary Information

**Supplementary Material 1.**

## Data Availability

No datasets were generated or analysed during the current study.

## Abbreviations

AMPK: Adenosine monophosphate-activated protein kinase
LIHC: Liver hepatocellular carcinoma
TCGA: The Cancer Genome Atlas
MHC-I: Major histocompatibility complex class I
TME: The tumor microenvironment
GTEx: Genotype-Tissue expression
scRNA-seq: Single-cell RNA-seq
CNGBdb: China National Genebank Database
GSCA: Gene set cancer analysis
t-SNE: t-distributed stochastic neighbor embedding
GO: Gene Ontology
KEGG: Kyoto Encyclopedia of Genes and Genomes
FBS: Fetal bovine serum
shRNA: Short hairpin RNA
qPCR: Quantitative real-time PCR
GSVA: Gene set variation analysis
EMT: Epithelial–mesenchymal transition
IFN-γ: Interferon-gamma
TCA cycle: Citrate cycle
